# Supplementary material for: Dynamic transcriptomic profiles of zebrafish gills in response to zinc supplementation
Source: BMC Genomics. 2010 Oct 11;11:553. doi: 10.1186/1471-2164-11-553 (PMC3091702; doi:10.1186/1471-2164-11-553)
Supplement: Additional file 2 — Interactive Direct Interaction Network representing the molecular interactions between zinc, copper, iron, calcium and proteins encoded by transcripts changed by zinc supplementation. Mini web-site containing index.html and hyperlinked pages in subdirectory describing a Direct Interaction Network automatically generated based on curated interactions contained within the proprietary PathwayArchitect database. Ovals represent proteins and the circles symbolize metal ions. Objects are coloured by their abundance in zebrafish at the time-point they were significantly different from the control is a scale from -4 fold (dark green) to +4 fold (dark red). Where significant differences were found at more than one time-point, the colour overlay shows expression at the first instance. Dark blue squares denote 'binding', and light blue squares 'expression'; green squares stand for 'regulation', green diamonds for 'metabolism', and green circles for 'promoter binding'. Arrow heads indicate directionality of the interaction where annotated. All nodes and edges can be further interrogated by selecting the relative area of the image. [file 1471-2164-11-553-S2.zip › PathwayArchitect Zn xs DIN/519086.html]

# REGULATION:

|  |  |
| --- | --- |
| Type | REGULATION |
| Effect | Positive |


---

|  |  |
| --- | --- |
| Score | 0 |


---

|  |  |
| --- | --- |
| Reference Count | 16 |


---

|  |  |
| --- | --- |
| Mechanism | Unknown |


---

|  |  |
| --- | --- |
| Reference:0 || Sentence | "The initial step of the activating mechanism involves association of the activator with calpain, followed by autoproteolytic activation of the proteinase in the presence of 1 microM Ca2+, at a rate identical to that induced by 1 mM Ca2+." |
| PMID | 8439288 |
| Year | 1993 |
| Species | Human |
| Journal | Biochem J |
| RefScore | 2 |
| Source | PArchNLP |
  |
|  |  |


---

|  |  |
| --- | --- |
 Reference:1 || Sentence | "These results suggest that cleavage of Htt in human HD tissue is mediated in part by the Ca2+-activated neutral protease, calpain." |
| PMID | 12077181 |
| Year | 2002 |
| Species | Human |
| Journal | J Neurosci |
| RefScore | 2 |
| Source | PArchNLP |
  ||  |  |


---

|  |  |
| --- | --- |
 Reference:2 || Sentence | "Aberrant Ca(2+) homeostasis following SCI inevitably activates calpain, which has been shown to play a crucial role in the pathophysiology of SCI." |
| PMID | 12738057 |
| Year | 2003 |
| Species | Human |
| Journal | Brain Res Brain Res Rev |
| RefScore | 0 |
| Source | PArchNLP |
  ||  |  |


---

|  |  |
| --- | --- |
 Reference:3 || Sentence | "It is unclear whether Ca(2+)-induced calpain activation is followed by subunit dissociation or not." |
| PMID | 12591934 |
| Year | 2003 |
| Species | Human |
| Journal | J Biol Chem |
| RefScore | 1 |
| Source | PArchNLP |
  ||  |  |


---

|  |  |
| --- | --- |
 Reference:4 || Sentence | "Conversely, excess extracellular Ca2+ entered Ca2+ channels and increased intracellular Ca2+ leading to calpain activation and necrosis." |
| PMID | 10217261 |
| Year | 1999 |
| Species | Human |
| Journal | J Neurochem |
| RefScore | 0 |
| Source | PArchNLP |
  ||  |  |


---

|  |  |
| --- | --- |
 Reference:5 || Sentence | "This leakage may reflect degradation of membrane proteins by the Ca2+-activated neutral protease calpain." |
| PMID | 11104058 |
| Year | 2000 |
| Species | Human |
| Journal | Eur J Appl Physiol |
| RefScore | 1 |
| Source | PArchNLP |
  ||  |  |


---

|  |  |
| --- | --- |
 Reference:6 || Sentence | "However, the concentrations of Ca2+ required to activate calpain are higher than those attained in the cytoplasm of most cells." |
| PMID | 8449989 |
| Year | 1993 |
| Species | Human |
| Journal | J Cell Biol |
| RefScore | 2 |
| Source | PArchNLP |
  ||  |  |


---

|  |  |
| --- | --- |
 Reference:7 || Sentence | "The mechanisms responsible for the above processes were investigated using E-64-d, a specific membrane-permeable inhibitor of Ca(2+)-activated protease (calpain); tetracaine, an activator of calpain; and N-ethylmaleimide and diamide, two sulfhydryl-reactive agents." |
| PMID | 8490169 |
| Year | 1993 |
| Species | Human |
| Journal | Blood |
| RefScore | 2 |
| Source | PArchNLP |
  ||  |  |


---

|  |  |
| --- | --- |
 Reference:8 || Sentence | "A comparative study of the susceptibility of vimentin and nuclear lamins from cultured Ehrlich ascites tumor (EAT) cells to degradation by Ca2+ -activated neutral thiol proteinase (calpain) has been undertaken." |
| PMID | 2846305 |
| Year | 1988 |
| Species | Human |
| Journal | Eur J Cell Biol |
| RefScore | 1 |
| Source | PArchNLP |
  ||  |  |


---

|  |  |
| --- | --- |
 Reference:9 || Sentence | "Here we present evidence for the posttranslational regulation of p107 by the Ca2+-activated protease calpain." |
| PMID | 10086333 |
| Year | 1999 |
| Species | Human |
| Journal | Oncogene |
| RefScore | 0 |
| Source | PArchNLP |
  ||  |  |


---

|  |  |
| --- | --- |
 Reference:10 || Sentence | "The association of the Ca(2+)-activated neutral protease, calpain, with membrane proteins following hydrazine insult was also examined using immunoblot analysis." |
| PMID | 1949012 |
| Year | 1991 |
| Species | Human |
| Journal | Toxicol Appl Pharmacol |
| RefScore | 1 |
| Source | PArchNLP |
  ||  |  |


---

|  |  |
| --- | --- |
 Reference:11 || Sentence | "Spinal cord injury (SCI) evokes an increase in intracellular free Ca(2+) level resulting in activation of calpain, a Ca(2+)-dependent cysteine protease, which cleaves many cytoskeletal and myelin proteins." |
| PMID | 12738057 |
| Year | 2003 |
| Species | Human |
| Journal | Brain Res Brain Res Rev |
| RefScore | 1 |
| Source | PArchNLP |
  ||  |  |


---

|  |  |
| --- | --- |
 Reference:12 || Sentence | "Following exposure to microM Ca2+, membrane-bound 80 kDa calpain is converted to the 78 kDa form, this conversion being linearly correlated with the expression of the proteinase activity." |
| PMID | 8769305 |
| Year | 1996 |
| Species | Human |
| Journal | FEBS Lett |
| RefScore | 1 |
| Source | PArchNLP |
  ||  |  |


---

|  |  |
| --- | --- |
 Reference:13 || Sentence | "Finally intracellular Ca(2+) fluxes activate the Ca(2+) requiring enzyme, calpain, which regulates cluster formation of leucocyte integrins." |
| PMID | 11108953 |
| Year | 2000 |
| Species | Human |
| Journal | Biochim Biophys Acta |
| RefScore | 1 |
| Source | PArchNLP |
  ||  |  |


---

|  |  |
| --- | --- |
 Reference:14 || Sentence | "Our results clearly show (i) that aminophospholipid exposure and platelet microvesiculation are independent but closely regulated events and (ii) that while both processes are associated with an increase in intracellular Ca2+, microvesiculation additionally requires Ca(2+)-induced calpain activation and a fusion process inhibited by annexin V." |
| PMID | 7547894 |
| Year | 1995 |
| Species | Human |
| Journal | Biochemistry |
| RefScore | 2 |
| Source | PArchNLP |
  ||  |  |


---

|  |  |
| --- | --- |
 Reference:15 || Sentence | "Calpains are non-lysosomal cysteine proteases that are Ca+2 activated." |
| PMID | 9768434 |
| Year | 1998 |
| Species | Human |
| Journal | Ren Fail |
| RefScore | 1 |
| Source | PArchNLP |
  |


---

|  |  |
| --- | --- |
